# Winners and losers of land use change: A systematic review of interactions between the world’s crane species (*Gruidae*) and the agricultural sector

**DOI:** 10.1002/ece3.8719

**Published:** 2022-03-24

**Authors:** Karoline Hemminger, Hannes König, Johan Månsson, Sonoko‐Dorothea Bellingrath‐Kimura, Lovisa Nilsson

**Affiliations:** ^1^ Leibniz‐Centre for Agricultural Landscape Research (ZALF) Müncheberg Germany; ^2^ Humboldt‐Universität Berlin Berlin Germany; ^3^ Grimsö Wildlife Research Station Department of Ecology Swedish University of Agricultural Sciences Riddarhyttan Sweden

**Keywords:** coexistence, conservation conflict, crop damage prevention, crop protection, human–wildlife conflict, human–wildlife interaction, land sharing, land sparing

## Abstract

While agricultural intensification and expansion are major factors driving loss and degradation of natural habitat and species decline, some wildlife species also benefit from agriculturally managed habitats. This may lead to high population densities with impacts on both human livelihoods and wildlife conservation. Cranes are a group of 15 species worldwide, affected both negatively and positively by agricultural practices. While eleven species face critical population declines, numbers of common cranes (*Grus grus*) and sandhill cranes (*Grus canadensis*) have increased drastically in the last 40 years. Their increase is associated with higher incidences of crane foraging on agricultural crops, causing financial losses to farmers. Our aim was to synthesize scientific knowledge on the bilateral effects of land use change and crane populations. We conducted a systematic literature review of peer‐reviewed publications on agriculture‐crane interactions (*n* = 135) and on the importance of agricultural crops in the diet of cranes (*n* = 81). Agricultural crops constitute a considerable part of the diet of all crane species (average of 37%, most frequently maize (*Zea mays* L.) and wheat (*Triticum aestivum* L.)). Crop damage was identified in only 10% of all agriculture‐crane interactions, although one‐third of interactions included cranes foraging on cropland. Using a conceptual framework analysis, we identified two major pathways in agriculture‐crane interactions: (1) habitat loss with negative effects on crane species dependent on specific habitats, and (2) expanding agricultural habitats with superabundant food availability beneficial for opportunistic crane species. The degree to which crane species can adapt to agricultural land use changes may be an important factor explaining their population response. We conclude that multi‐objective management needs to combine land sparing and land sharing strategies at landscape scale. To support viable crane populations while guaranteeing sustainable agricultural production, it is necessary to include the perspectives of diverse stakeholders and streamline conservation initiatives and agricultural policy accordingly.

## Graphical abstract

While eleven crane species face critical population declines, the drastic increase in common cranes (*Grus grus*) and sandhill cranes (*Grus canadensis*) is associated with higher incidences of cranes damaging agricultural crops. We conducted a systematic literature review of peer‐reviewed publications on agriculture‐crane interactions (*n* = 135) and on the importance of agricultural crops in the diet of cranes (*n* = 81). A conceptual framework identified two major pathways: (1) habitat loss with negative effects on crane species dependent on specific habitats, and (2) expanding agricultural habitats with superabundant food availability beneficial for opportunistic crane species.

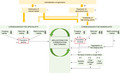

## 1 INTRODUCTION

Global human population growth contributes to rising natural resource needs, which accelerates the intensification and expansion of agricultural production. However, intensification of agriculture and the associated use of pesticides and decrease in crop varieties are associated with a decline in biodiversity (Emmerson et al., 2016; Flynn et al., 2009; Kleijn et al., 2009). The negative consequences of agricultural intensification are further exacerbated by the expansion of agricultural areas, which leads to the loss of natural habitats and fragmentation (Balmford et al., 2012; Dobrovolski et al., 2011; Foley et al., 2005). Therefore, one of today's major challenges is to combine the objectives of food security and biodiversity conservation. While acknowledging the alarming trends of biodiversity decline, land use change is not necessarily negative for all species. Species that are able to use habitats within large‐scale agriculture can even benefit from landscape fragmentation. Because of this difference in adaptability among species, land use change can have effects on species composition and may increase species density in remnant patches of natural habitat (Devictor et al., 2008; Tittonell et al., 2020; Tscharntke et al., 2012). In this way, expansion of agricultural area at the expense of natural habitat likely contributes to higher frequencies of human–wildlife interactions (Jochum et al., 2014; König et al., 2020). These interactions can have negative consequences for both humans and wildlife. Farmers face losses caused by crop damage or livestock killing by wildlife, while conservation efforts may be negatively affected when wildlife is disturbed, poached, or poisoned as a means of preventing damage to human livelihoods (Redpath et al., 2013; Seoraj‐Pillai & Pillay, 2017). Therefore, it is crucial to increase knowledge of the mechanisms underlying the contrasting responses of species to agricultural activities. Why do certain species become “winners” and others “losers”? Such knowledge can help to find compromises between objectives of agricultural production and biodiversity conservation at landscape scale and thus alleviate conservation conflicts (Redpath et al., 2013).

Agricultural expansion and intensification have been identified as the leading threat to 74% of the world's bird species, and at least 40% of all bird species exhibit declining population trajectories (Bird Life International, 2018). For example, bird populations in North America and Europe have declined by at least 30% since the 1970s, with a particularly steep decline in farmland birds (Chamberlain et al., 2000; Donal et al., 2001; Inger et al., 2015; Rosenberg et al., 2019). Cranes (*Gruidae*) are a family of 15 bird species distributed globally and have been both negatively and positively affected by agricultural practices (Austin et al., 2018). Most crane species rely on shallow wetlands for night roosting and nesting. However, their foraging habitat includes cropland as well as pastures, and cranes’ diurnal flights to foraging areas are an example of cross‐habitat movement that connects patches of agricultural area and natural habitat (Prange, 2016; Tscharntke et al., 2012). Moreover, most cranes are migratory and use several staging sites along their flyways (Krapu et al., 2014). Conservation strategies thus need to be coordinated among countries covered by the flyways. Eleven crane species face critical population declines and are classified by the IUCN as vulnerable, endangered, or critically endangered. In contrast, four crane species are currently increasing in number (Mirande & Harris, 2019). The population increase has been particularly evident for sandhill cranes (*Antigone canadensis* Linnaeus) and common cranes (*Grus grus* Linnaeus) in the last 40 years, with populations reaching sizes above 800,000 and 700,000 individuals, respectively (Bird Life International, 2016; Krapu et al., 2019; Nagy et al., 2014; Prange & Ilyashenko, 2019). While the recovery of these species can be viewed as conservation success, the likelihood of cranes foraging in agricultural areas has increased and is associated with a higher risk of crop damage and financial losses for farmers (Alonso et al., 2018; Andryushenko, 2018; Austin, 2018; Barzen et al., 2018; Lacy, 2018; Montràs‐Janer et al., 2019; Salvi, 2010; Shanni et al., 2018; Végvári et al., 2010). A recent IUCN publication on cranes and agriculture (Austin et al., 2018) identifies the global importance of this topic and reports that vulnerable crane species such as gray‐crowned cranes (*Balearica regulorum* Bennet) or blue cranes (*Anthropoides paradiseus* Lichtenstein) may also cause crop damage (Fakaryi et al., 2018; van Niekerk, 2018). Knowledge of cranes’ foraging behavior in agricultural landscapes is thus of great interest for agriculture and conservation in order to improve crop protection and the possibility of coexistence in agricultural landscapes. Moreover, insights into the importance of agricultural crops for crane diets can help secure essential foraging habitats. The aim of this review was to contribute to a better understanding of the bilateral effects of land use changes by the agricultural sector and the behavior and population trajectories of crane species by answering the following research questions:

1. What is the state of scientific knowledge on interactions between agriculture and cranes (section III.1)?
2. How do crane species use agricultural areas for foraging (section III.2)?
3. How can knowledge of agriculture‐crane interactions be integrated to inform multi‐objective management of crane populations and agricultural production (section III.3)?

The synthesized knowledge will be used to identify differences in response to land use change among crane species (section IV.1), recommend management implications to sustain both agricultural production and crane conservation (section IV.2), and identify important research gaps (section IV.3).

## 2 METHODS

The analysis included two parts. The first part constituted a systematic literature review and conceptual framework analysis of agriculture‐crane interactions. We searched peer‐reviewed literature in Scopus and Web of Science databases using all combinations of scientific names (“*grus*,*”* “*gruidae*,*”* “*balearica*,*”* “*anthropoides*,” “*bugeranus*,*”* “*antigone")*, English names (*“siberian crane*,*" "grey crowned crane*,*" "red*‐*crowned crane*,*" "whooping crane*,*" "blue crane*,*" "black crowned crane*,*" "hooded crane*,*” "sarus crane*,*”* *"wattled crane*,*"* *"white naped crane*,*" "brolga*,*" "demoiselle crane*,*" "eurasian crane*,*" "common crane*,*""sandhill crane"*) and the terms “farm,” “agri*,” and “land use.” These terms were initially discussed and selected by the authors to find information on the leading question “What is the scientific evidence on interactions between agriculture and cranes?” We restricted the bibliographic analysis to original research articles in peer‐reviewed journals published from 1999 to 2019. In Scopus, the subject areas “engineering” and “computing” were excluded; for the search in Web of Science, these subject areas were sorted out manually because of the homonym “crane” (machine). After the exclusion of review articles, the search criteria for agriculture‐crane interactions resulted in 178 original research articles. In a second step, the following articles were excluded: articles selected based on homonyms in the abstract and keywords (crane, wind farm, GRUs, farm animals, and crane fly (*n* = 32)) and articles containing no information on our definition of agriculture‐crane interactions (i.e., no species‐specific information, articles on cranes in captivity, and articles on bird strikes in aviation or avian hepatitis (*n* = 12)), resulting in a final list of 135 peer‐reviewed publications. To integrate the information in the selected articles, we built on a research method for conceptual analysis described by Jabareen (2008). In the first phase, we analyzed the content of each article to identify the interactions between agriculture and each crane species. In a second phase, we identified patterns within the sample of articles and created a list of possible interactions for each crane species. In the third phase, we merged similar interactions to create 19 main interactions. These interactions were then classified into four main categories of impact and response. Each publication was assigned to one of these categories based on its research focus. Phase four constituted the building of a qualitative conceptual model describing the relationships among the derived categories. Conceptual models have been defined as abstract representations of reality. They can be used to integrate information from several scientific disciplines to ease the understanding of complex systems. Qualitative modeling is a tool used to structure and construct knowledge by visualizing directions of influence for relationships that cannot be mathematically defined (Fortuin et al., 2011).

The second part of our research was a systematic literature review of the diet composition of cranes. Using the same procedure as for the first part of the analysis, we searched the above‐mentioned databases for the above‐mentioned taxonomic terms in combination with the terms “nutrition,” “forag*,” “feces,” “feed,” “resource,” “diet,” and food*.” These terms were discussed and selected by the team of authors to find information on research question 2 (“How do cranes use agricultural areas for foraging?"). We used the quantitative measure of diet composition as an indicator of how well different crane species can adapt to forage on cropland. In Scopus, the subject areas “engineering” and “computing” were excluded, and in Web of Science, these subject areas were excluded manually. The following inclusion criteria for articles were used: (i) peer‐reviewed original research articles, (ii) information on crane diet composition, and (iii) published between 1999 and 2019. After exclusion of review articles and articles found based on homonyms, we created a list of 586 original research articles. Due to the variety of search terms, a majority of the articles (*n* = 516) did not contain information on crane diet and were excluded manually (i.e., topics that were excluded ranged from crane habitat, behavior, and conservation to microbiology). The 51 remaining articles were screened a second time with the following inclusion criteria: (i) quantitative information on crane diet composition and (ii) methods of stomach content analysis, stable isotope analysis, fecal analysis, or field observation of diet and ingestion. As only 13 articles fit the search criteria, the time horizon was increased to 1970–2019, and articles were screened using the same procedure as described above, resulting in 61 publications with qualitative information and 20 publications with quantitative information on crane diet composition. Some articles contained diet composition data for the staging, wintering, and breeding seasons or data collected at more than one site. In such cases, we chose to display all data separately to create factors for intraspecific variation in diet composition, which were more visible (in the end, 37 results were displayed for 20 publications). Weight and volume data were converted to the percentage of crops, other plant‐derived food, and animal‐derived food in the diet. To complement the quantitative results, we performed a topical review of the remaining 61 articles.

Based on FAO terminology, the following definitions for land use are used throughout the article: Agricultural area = land area mainly devoted to agriculture, including cropland and permanent grassland. Cropland = all land used for cultivation of annual (e.g., cereals) and permanent crops (e.g., fruit trees) including paddy fields. Permanent grassland = all land used permanently (more than five years) to grow herbaceous forage crops through cultivation or naturally (wild prairy or grazing land).

## 3 RESULTS

### 3.1 Agriculture‐crane interactions

Definition of agriculture‐crane interactions and number of corresponding articles selected for analysis in this paper.

**FIGURE 1:**
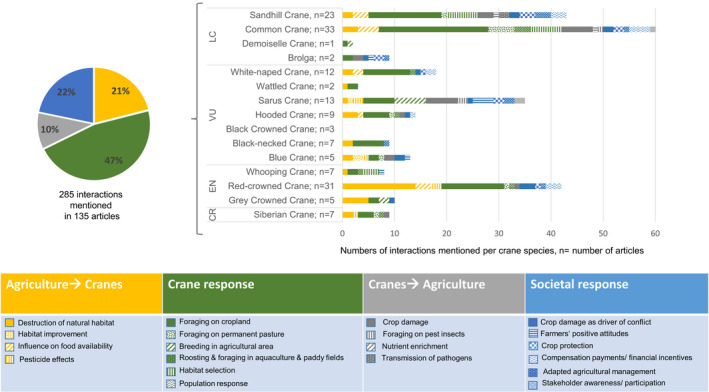
Overview of agriculture‐crane interactions identified in the reviewed articles and categorization of agricultural impacts on cranes (orange), crane impacts on agriculture (gray), crane responses (green), and societal responses (blue). Crane species are sorted based on the categorization of the IUCN Red List for endangered species: LC = least concern, VU = vulnerable, EN = endangered, CR = critically endangered

Based on the 19 agriculture‐crane interactions found in our analysis (Figure 1), we concluded that the interactions can lead to two‐way effects on cranes and humans. Agriculture and related land use can affect crane habitat and food availability (category 1, Figure 1), but cranes can also negatively affect agricultural production (category 2, Figure 1). These two‐way effects can in turn lead to various responses, that is, cranes potentially adapting to changes in agriculture and land use (category 3, Figure 1). Humans respond to crane foraging by changing agricultural practices and by investing in crop damage prevention or compensatory strategies (category 4, Figure 1). Most available publications focused on the behavior of cranes, whereas only a few publications covered the effect of cranes on agriculture or responses of farmers and strategies to mitigate effects (Table 1). Agriculture‐crane interactions were identified for all species except black‐crowned cranes. In accordance with the focus of most articles, almost half of the identified interactions described crane behavior from an animal ecology perspective (e.g., foraging behavior and habitat selection, depicted in green in Figure 1).

**TABLE 1:** Definition of agriculture‐crane interactions and number of corresponding articles selected for analysis in this paper

	Category	Inclusion criteria	No. articles in review
Effect	1. Agriculture's effect on cranes	Information on land use and habitat change over time, effect of pesticides on cranes, effect of agricultural management on forage availability	21 (15.7%)
	2. Crane´s effect on agriculture	Information on crop damage and other negative and positive effects of cranes on farming	10 (7.5%)
Response	3. Adaptation of cranes to habitat changes	Information on crane habitat selection, foraging and migration pattern, behaviour and population numbers	77 (57.5%)
	4. Societal Response	Information on farmer attitudes towards cranes and options for management of cranes on agricultural land	13 (9.7%)
	Several Categories	Combination of information from categories 1–4	13 (9.7%)

The majority of publications focused on common cranes (24%), red‐crowned cranes (*Grus japonensis* Statius Müller) (23%), and sandhill cranes (17%). The largest share of publications on common cranes focused on habitat selection, space use, and the effect of land use change on population numbers (46%). In comparison, more publications on sandhill cranes focused on interactions with agriculture, such as agricultural extension, diversionary feeding, and the influence of agricultural practices on forage availability (26%), whereas the largest share of publications on red‐crowned cranes addressed the effect of habitat changes on population numbers (19%). The highest number of interactions in the categories of cranes’ impact on agriculture and societal response were identified for the sarus crane (*Antigone antigone* Linnaeus) (19%; e.g., effect of crane nests in fields on rice harvest *(Oryza sativa* Linnaeus) or the positive attitude of farmers toward cranes) (Figure 1). Below, each of the identified agriculture‐crane interactions will be described. For a summary of all agriculture‐crane interactions, the crane species for which each interaction was identified and key references, see Appendix S2.

### 3.2 Effect of agriculture on cranes

#### 3.2.1 Destruction of natural habitat

Agricultural land use change as a driver of wetland degradation was reported for 12 out of 15 species (Appendix S2) and constituted 15% of all agriculture‐crane interactions (Figure 1). For example, sandhill cranes abandoned traditional wintering sites in wetland prairies when natural wetlands were converted to cropland (Taft & Haig, 2003). Similarly, farmland expansion in China contributed to the degradation of wetlands in several breeding and staging sites of red‐crowned cranes (Han et al., 2007; Ke et al., 2011; Li et al., 2012; Wang et al., 2003, 2014). Moreover, hooded cranes (*Grus monacha* Temminck) were observed to spend less time foraging in wetlands degraded by aquaculture than in natural wetlands, which indicates lower food availability in wetlands with aquaculture (Zhou et al., 2010).

#### 3.2.2 Habitat improvement

Agricultural expansion can also lead to the creation of new habitat for cranes. This is the case when natural habitats that are not suitable for a certain crane species are converted to agricultural area. In South Africa, the conversion of natural shrubland to cereal fields and permanent pastures led to an increasing number of foraging and breeding blue cranes. These cranes showed higher survival rates than a population using natural habitats (McCann et al., 2007; Van Velden et al., 2017). Similarly, sarus cranes in Australia expanded their distribution to new areas after natural eucalypt forest and rainforest were destroyed to create cropland that could be used by the cranes (Nevard et al., 2019).

#### 3.2.3 Influence on the availability of food for cranes in agricultural areas

Inter‐ and intra‐annual dynamics of cultivated crops and agricultural practices influence food availability for cranes in agricultural areas. These dynamics depend on economic incentives, such as world market prices and subsidies, as well as the development of agricultural technology, crops, pesticides, and fertilizers (FAO, 2020; Mesterházy et al., 2020). For example, a change in commonly used crops in an area can significantly change food availability for cranes. In the case of sandhill cranes, a shift from cultivation of maize to soybeans resulted in larger patches of habitats of limited or no value, which caused greater flight distances to foraging sites and reduced the body fat storage of cranes (Krapu et al., 2004, 2014; Pearse et al., 2010, 2016). Similarly, sarus cranes were negatively affected when farmers in Nepal and Australia shifted from cereals to sugar cane and tree crops due to a change in market prices (Aryal et al., 2009; Nevard et al., 2018). The availability of food for cranes is also influenced by the timing of agricultural practices. For example, earlier harvests in response to warmer climate have decreased the availability of stubble fields for autumn staging common cranes in Hungary (Vegvari, 2002). Food availability is also dependent on the timing and type of tillage. Whereas plowing buries residual grains and plants, mulching cuts leftover plant material, leaving residual grain on the soil surface (Krapu et al., 2004). Technical development of harvesting machines increases harvest efficiency and thus reduces the amount of residual grain (Anteau et al., 2011; Krapu et al., 2004; Pearse et al., 2010; Sherfy et al., 2011). The influence of agricultural practices on food availability in paddy fields is, however, not well studied. Leaving paddy fields unplowed throughout winter is recommended to enhance foraging opportunities for red‐crowned and white‐naped cranes (*Antigone vipio* Pallas) in China and South Korea (Lee et al., 2007; Lu et al., 2006), whereas wintering common cranes have been observed foraging in plowed paddy fields in Spain (Guzmán et al., 1999). We found no information on the influence of agricultural practices on the availability of animal and insect prey in agricultural areas (Nowald & Fleckstein, 2001).

#### 3.2.4 Pesticides

Cases of poisoning by agricultural pesticides have been reported for red‐crowned cranes, sarus cranes, and blue cranes (Borad et al., 2002; Botha et al., 2015; Lu et al., 2007; Mukherjee‐Wilske et al., 2002; Trigunayat, 2003; Van Velden et al., 2017). Some cases happened incidentally (e.g., cranes foraged on seeds treated against other pests), and in some cases, farmers purposely targeted cranes (e.g., used pesticides to prevent crop damage or poisoned baits to kill them). Pesticides may also have indirect effects on cranes, such as when herbicides diminish the availability of weed seeds available for foraging (Krapu et al., 2004) or when excessive use of fertilizers and pesticides leads to contamination of waterways and effects on the aquatic prey of cranes (Jinming et al., 2017).

### 3.3 Crane response

In line with the focus of most publications, almost half of the interactions (47%) concerned the opportunistic behavior of cranes (Figure 1). The most frequently (32%) described form of interaction with agriculture was “foraging on cropland.”

#### 3.3.1 Foraging in agricultural areas

Cranes forage both in permanent pastures and on cropland. Permanent pastures are a typical foraging habitat for several crane species. Holm oak dehesas, a traditional system of pig grazing in Spain, are foraging sites for wintering common cranes (Avilés, 2003; Franco et al., 2000). Additionally, blue cranes, demoiselle cranes (*Grus virgo* Linnaeus), hooded cranes, sandhill cranes, and Siberian cranes *(Leucogeranus leucogeranus* Pallas) forage in permanent pastures (see Appendix S2 and Figure 1). Foraging on cropland was described for all crane species except for black‐crowned cranes (*Balearica pavonina* Linnaeus) (Appendix S2, Figure 1). However, the extent to which each species forages in agricultural areas greatly differs between species (see section III.2 below), and cranes were found to both select and avoid agricultural areas. Blue cranes, common cranes, and gray‐crowned cranes (*Balearica regulorum* Bennet) selected agricultural areas over natural habitats for foraging (Fakarayi et al., 2016; Nilsson et al., 2019; Van Velden et al., 2016). As cranes rely on shallow wetlands for night roosting, fields near wetlands have a higher probability of being used by cranes (Anteau et al., 2011; Nilsson et al., 2018). Few of the analyzed studies differentiated between crane use of agricultural areas based on the types and stages of crops or the size of fields. For example, autumn staging common cranes primarily foraged on maize stubble fields rather than other cultivated fields in Hungary (Vegvari, 2002) and have also been shown to select stubble fields over unharvested crops and bare soil in Sweden (Nilsson et al., 2016). Similarly, sandhill cranes used mulched over plowed fields in Nebraska (Anteau et al., 2011) and foraged more frequently in areas dominated by maize compared with soybean cultivation (Krapu et al., 2004). A study on sandhill cranes in Nebraska revealed that crane use of newly sown maize fields was high the first 25 days after germination but decreased afterward (Barzen et al., 2020). Responses to different types of agricultural management varied among crane species. For example, in Zimbabwe, wattled cranes (*Grus caranculata* Gmelin) were associated with large‐scale farms with less human disturbance, whereas gray‐crowned cranes were associated with small‐scale farms and communal grazing land. The crop chufa (*Cyperus esculentus* L.) and bare ground positively influenced the abundance of both crane species, but the influence was weaker for gray‐crowned cranes than for wattled cranes (Fakarayi et al., 2016). Avoidance of agricultural areas was also found, for example, Siberian cranes selecting natural wetlands far from agricultural areas (Kong et al., 2013), wattled cranes breeding in areas dominated by natural grassland (McCann & Benn, 2006) and black‐necked cranes (*Grus nigricollis* Przhevalsky) using agricultural areas only when the availability of natural habitats was limited (Liu et al., 2010).

#### 3.3.2 Breeding in agricultural areas

A few crane species respond to land use change by breeding in agricultural areas. Stubble fields are the main nesting biotopes of Demoiselle cranes in Russia (Korovin, 2011). In India, sarus cranes are using bunds of rice fields as nest sites, although hatching success is lower in these sites than in natural marshland (Borad et al., 2002; Sundar, 2009).

#### 3.3.3 Roosting and foraging in aquaculture and rice paddy fields

Artificial wetlands such as aquaculture and rice paddy fields are used by cranes both for night roosts and for foraging (Fujioka et al., 2010; Wood et al., 2010). Many crane species regularly forage in rice paddy fields, such as described for common cranes (Guzmán et al., 1999), sarus cranes (Aryal et al., 2009; Borad et al., 2001a), red‐crowned cranes (Kim et al., 2016), or whooping cranes (North America: Pickens et al., 2017). The importance of fishponds as a roosting site during autumn staging is mentioned for common cranes in Hungary (Végvári & Barta, 2015). In China, the proportion of wintering red‐crowned cranes roosting in artificial wetlands increased from ~40% in 1982 to ~55% in 2005 (Lu et al., 2006), which points at cranes shifting to use artificial wetlands in response to destruction of natural wetlands.

#### 3.3.4 Site and habitat selection in response to agriculture

Agricultural practices can affect food availability and foraging site selection by cranes within staging sites (see section 1.b.i). However, agricultural practices may also affect crane migration strategies such as the timing and duration of staging. For whooping cranes (*Grus americana* Linnaeus), the extent and type of crops in an area was identified as one of several factors influencing their use of staging sites and possibly also contributing to changes in migration route (Belaire et al., 2014; Pearse et al., 2016, 2018). Similarly, the number of common cranes was higher at autumn staging sites with large shares of harvested winter cereal fields in the surroundings than at staging sites surrounded by fields with tilled spring barley (*Hordeum vulgare* L.), and the number of sandhill cranes increased at a wintering site when agriculture shifted from rain‐fed to high‐yielding irrigated maize (López‐Saut et al., 2011; Mireles & Mellink, 2017). Cranes may also change wintering sites in response to agricultural management. Common cranes used newly established paddy fields over holm oak dehesas, which resulted in prolonged wintering periods in Spain. The earlier peak of wintering cranes correlated with the highest availability of acorns (Guzmán et al., 1999). In contrast, black‐necked cranes abandoned a former wintering site in Bhutan when potatoes (*Solanum tuberosum* L.) were planted instead of the traditional winter fallow (Lhendup & Webb, 2009).

#### 3.3.5 Population development in relation to food availability

In addition to other factors, food availability derived from agriculture is seen as an important driver of population growth for some crane species (Krapu et al., 2004; Leito et al., 2008; Lu et al., 2007). Several studies indicate a reciprocal effect between the availability of agricultural food and population numbers: increased numbers of sarus and blue cranes are linked to a shift in agricultural production that increased the availability of residual grain in Australia and South Africa (McCann et al., 2007; Van Velden et al., 2017). The availability of high‐energy foods (i.e., mostly residual maize) was also identified as important factor for the accumulation of body fat by sandhill cranes during spring staging influencing both their survival rates during migration and reproduction success (Krapu et al., 2014). In contrast, a decline in the availability of residual grain may also negatively affect cranes, as observed for demoiselle cranes in the Trans‐Ural steppe (Korovin, 2011). Interestingly, nest survival of sandhill cranes was not affected by the type of management of pastures in the surroundings (i.e., unused vs. grazed pasture; Austin et al., 2007).

### 3.4 Effect of cranes on agriculture

#### 3.4.1 Crop damage

Crop damage constituted less than 10% of all agriculture‐crane interactions, although 32% of all interactions involved cranes “foraging on cropland” (Figure 1). Nineteen studies referred to crop damage but did not present crop damage data. The exceptions were two publications from Sweden, in which crop damage by cranes was assessed by a standardized protocol for governmental compensation payments (Cusack et al., 2019; Montràs‐Janer et al., 2019). Assessed across Sweden, crop damage was found to be linearly related to the number of common cranes. The crops most often reported to be damaged by cranes were barley, wheat (*Triticum aestivum* L.), potatoes, legumes (*Fabaceae* spp.), and carrots (*Daucus carota* subsp. *Sativus* Hoffm.; Montràs‐Janer et al., 2019 *)*. A study on sandhill cranes showed that crane use of maize fields decreased seedling density (Barzen et al., 2020). Three publications used farmer interviews or surveys to analyze the extent of crop damage, although such studies can provide important insights. Based on a farmer survey (*n* = 311), damage to crops by red‐crowned cranes was estimated to affect 2%–3% of the cultivated area at a wintering site in China (Bennett et al., 2018), and in Australia, 22 out of 31 interviewed farmers reported crop damage by sarus cranes and brolga, with most damage reported for peanuts followed by maize and the most critical periods for damage reported after sowing and just before or during harvest (Nevard et al., 2018). In South Africa, farmer interviews (*n* = 40) revealed regional differences in crop damage by blue cranes, with 65% of Swartland farmers reporting damages to sweet lupin, canola, and wheat at the early leaf stages, whereas farmers in the Overberg, a grassland‐dominated area, did not perceive that cranes caused damage (Van Velden et al., 2016).

#### 3.4.2 Foraging on pest insects

There are two records of cranes foraging on insects that can become a pest to agricultural crops: Sarus cranes foraging on pod borers (larvae of *Helicoverpa armigera* Hubner) in chickpea crops in India (Singh & Rizvi, 2010) and common cranes feeding on larvae of the silver Y (*Autographa gamma* Linnaeus) in maize crops in Germany (Nowald & Fleckstein, 2001).

#### 3.4.3 Effects on nutrient cycles

Two studies analyzed the fertilizing effect of crane foraging on agricultural fields. The amount of crane feces in paddy fields equaled 1 kg N/ha/year and 0.2 kg P/ha/year at a wintering site of common cranes in Spain (Navedo et al., 2015). At another common crane wintering site in Israel, the inflow of P to agricultural soil was even higher (~7 kg P/ha/year compared to ~6.4 kg P/ha/year removal by plant harvesting measured on the scale of a feeding site of ~100 ha) (Litaor et al., 2014). While nutrients added to soils by cranes are partly balanced by plant harvesting, the impact on waterways may be more problematic, as was also found at the wintering sites in Spain and Israel (Litaor et al., 2016a; Navedo et al., 2015).

#### 3.4.4 Transmission of pathogens

As migratory species, cranes are potential carriers of pathogens over extensive distances. This is particularly problematic when crane excrements act as a vector to infect agricultural products. For example, sandhill crane excrements on peas led to an outbreak of *Campylobacter* in humans in North America (Gardner et al., 2011). Black‐crowned cranes were reported to be infected with avian influenza virus. This points at cranes’ susceptibility to the disease, which may pose a risk for transmission between cranes and poultry farms (Bello et al., 2008).

### 3.5 Societal response

#### 3.5.1 Crop damage as a driver of conflict

Only 9% of the publications in our review included the term “conflict,” and stakeholders who were stated to be involved in the conflict varied (Table 2). The majority of these publications (75%) mentioned crop damage as a potential cause of conflict between stakeholders with conflicting objectives.

**TABLE 2:** Use of the term “conflict” in the analyzed body of articles on agriculture‐crane interactions

Example	Parties in conflict	No. Articles	References
“human‐wildlife conflict”; “human‐crane conflict”; “conflict between humans and cranes for access of food”	Humans vs. cranes	4	Olupot (2016), Van Velden et al. (2016), Van Velden et al. (2017), Mi et al. (2018)
“cranes in conflict with agricultural production”	Cranes vs. agricultural production	2	Nevard et al. (2019), Boggie et al. (2018)
“conflict between farming communities and nature reserve”	Stakeholders in agriculture vs. stakeholders in conservation	2	Nilsson et al. (2016), Bennett et al. (2018)
“conflicting ecological and economic interests”	Agricultural objectives vs. conservation objectives	4	Nilsson et al. (2019), Laubhan and Gammonley (2001), John et al. (2003), Kim et al. (2016)

Both assessments of the actual extent of crop damage (see section IV.3.a above) and assessment of the “conflict” are rare, and only two articles addressed farmer attitudes toward cranes. Among farmers in South Africa, only those with a high number of blue cranes on their fields during times of damage risk perceived cranes as problematic. Similarly, only 25% of US farmers who had observed sandhill cranes in their fields responded that “cranes conflicted with grain production.” In both cases, only farmers with high numbers of cranes in their fields during fall reported conflict (Laubhan & Gammonley, 2001; Van Velden et al., 2016).

#### 3.5.2 Positive attitudes of farmers toward cranes

In many cultures, cranes are symbols of luck and longevity and hold a prominent role in myths. This may also affect the way farmers interact with cranes. In Tibet, Uganda, and the Gangetic floodplains of India, traditional beliefs lead to people being considerate of cranes, and farmers protect nest sites within their fields (Che et al., 2018; Gopi Sundar, 2011; Muheebwa‐Muhoozi, 2001). These cultural beliefs are very specific to different locations. In contrast to those in the Gangetic floodplains, farmers in other parts of India reportedly persecute cranes, and chicks are collected for trade (Gopi Sundar, 2011).

#### 3.5.3 Crop protection

No comparative assessments of the effectiveness of the various methods to prevent crop damage by cranes were found in our review (for a detailed list of recommended methods, see (Austin et al., 2018; Austin & Sundar, 2018). Descriptions of hunting or poisoning cranes to prevent damage referred to single events (as described in section II.1.a.iv). Lure crops and diversionary feeding, that is, fields where the birds can feed undisturbed, have been used in Sweden (Montràs‐Janer et al., 2019), Israel (Litaor et al., 2016a), and North America (Boggie et al., 2018; Vogel et al., 2013). At an area in New Mexico in which diversionary feeding is practiced, as much as 60% of sandhill crane diet derived from supplemented maize (Boggie et al., 2018). Another method used in the United States for preventing sandhill cranes from consuming newly planted or sprouted seeds is to bait seeds with the post‐ingestive deterrent anthraquinone, which causes cranes to feel sick after consumption. It was shown that this method was effective in reducing the amount of maize seedlings damaged on fields used by cranes (Barzen et al., 2020).

#### 3.5.4 Compensation payments and financial incentives

Compensation payments are used as a strategy to manage conservation conflicts by reimbursing farmers for financial losses caused by wildlife damage (e.g., in India: Karanth et al., 2018; in Europe: Bautista et al., 2019). The reviewed articles did not contain information on how farmers’ acceptance of cranes was affected by compensation, and few publications report compensation costs, with an exception being costs of more than € 1 million between 2000 and 2015 in Sweden (Montràs‐Janer et al., 2019). Few publications have analyzed the potential use of a positive image of cranes for marketing agricultural products. Consumers were willing to pay only a marginal price premium for rice with a “crane friendly” label in Vietnam (Khai & Yabe, 2015), whereas a community conservation initiative in the Republic of Korea successfully used the positive image of cranes as a way to market rice at higher prices. Responding to this financial incentive, farmers shifted their previously negative perception and welcomed cranes to forage on their fields (John et al., 2003; Kim et al., 2011, 2016). A variety of articles argue that agricultural policy should provide financial incentives for practices that benefit cranes. Mentioned examples include cultivating a specific crop, adapting the timing of tillage to increase forage availability (Krapu et al., 2004; Sherfy et al., 2011), and maintaining and protecting suitable habitats, as typically found in traditional farming systems (Borad et al., 2001a, 2001b, 2002). Other publications point to the potential of crane staging sites for ecotourism with income opportunities that could be shared with farmers (Nevard et al., 2019; Robbins, 2003). Nevertheless, the concern is expressed that financial compensation to farmers is likely to dilute positive cultural attitudes toward cranes because once farmers become accustomed to receiving money to protect cranes, they might be less likely to continue crane protection without a payment (Sundar, 2009).

#### 3.5.5 Crane adapted agricultural practices

In addition to the financial interest of preventing crop damage, it is also acknowledged from a conservation perspective that agricultural areas should be maintained and protected as crane foraging sites. This can be achieved by adapting cultivation practices or by choosing crops that are selected by cranes (Liu et al., 2010; Vegvari, 2002). As previously described, the timing and type of tillage influence food availability for cranes (section II.1.a.iii above). However, only one study specifically examined agricultural practices used to increase food availability for cranes (Anteau et al., 2011). A change in the choice of cultivated crops could also be a strategy for avoiding crop damage by cranes. Farmers could change from crops with a high risk of damage by cranes to less vulnerable crops. However, only one study reported that affected farmers actually did this (Van Velden et al., 2016).

#### 3.5.6 Generating awareness and stakeholder participation

Cranes hold a positive cultural image, and in some locations, these beliefs are so strong that farmers will protect cranes on their land despite financial losses (Borad et al., 2002). It is, therefore, argued that using the importance of cultural habits to retain crane habitat is still overlooked in conservation strategies (Gopi Sundar, 2011). Accordingly, a number of articles recommend educational programs to improve residents’ awareness of the ecological and cultural value of cranes (Havrylenko, 2000; Katuwal, 2016; Kone et al., 2007; Muheebwa‐Muhoozi, 2001). Other articles recommend that conservation programs actively seek cooperation with farmers and other stakeholders who are affected by cranes (Mafabi, 2000; Nsengimana et al., 2019; Van Velden et al., 2017).

## 4 AGRICULTURAL CROPS IN THE DIET OF CRANES

We used the proportional share of agricultural crops in the diet as a measure of how the use of agricultural area varies among crane species. A literature search on the topic of crane diet composition revealed 15 publications covering seven different species, which quantified diet composition by stomach content analysis (*n* = 9), fecal analysis (*n* = 6), observation (*n* = 4), and stable isotope analysis (*n* = 1) (time span: 1970–2019). In these 15 publications, the proportion of crops in the diet of cranes averaged 37%. There is large inter‐ and intraspecific variation in the importance of crops, but all crane species have been documented to forage on crops (at least 5% of their diet, Table 3). We complemented this information with a review of the literature with qualitative descriptions of crane diets and found 81 studies in total. Most publications on diet covered sandhill cranes (*n* = 30) and red‐crowned cranes (*Grus japonensis* Müller) (*n* = 22); however, the overall data availability on crane diet composition was low, and for three species, neither quantitative nor qualitative information on diet composition was found (Table 3).

**TABLE 3:**
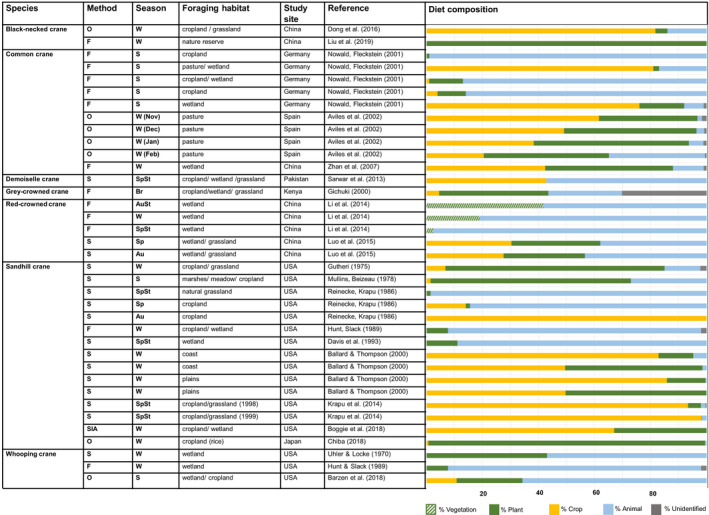
Overview of quantitative information on the diet composition of different crane species

### 4.1 Seasonal differences

Quantitative results show a higher importance of agricultural crops in the diet of crane species during wintering than during breeding and spring staging. We found a lower proportion of animal‐derived food during wintering (mean: 21%) than during breeding (50%) and spring staging (54%). This is an expected outcome, since cranes’ physiological need for animal‐derived protein is higher during the breeding period (common cranes: Nowald & Fleckstein, 2001; gray‐crowned cranes: Gichuki, 2000 and whooping cranes: Dinets, 2016; Nelson et al., 1996; Nelson et al., 1997). Moreover, crane behavior during breeding and chick rearing restricts their foraging habitat to areas directly surrounding their wetland breeding sites (Gichuki, 2000).

### 4.2 Species‐specific differences

Although all cranes share certain behavioral traits, each species has distinct physiological features that define the use of foraging habitats. For example, the bills of species adapted to dry grassland habitats (demoiselle and blue cranes) are much smaller than those of species primarily relying on wetland habitat (Siberian and whooping cranes), which allows the former to consume small insects and grass seeds, while the latter are better adapted to probe below ground for tubers and ground‐dwelling insects. The remaining eleven crane species forage in both wetland and non‐wetland habitats with species‐specific differences between breeding and non‐breeding seasons (see Table 4; Harris & Mirande, 2013; Harris & Mirande, 2013; Nowald et al., 2018; Prange, 2016, 2016). We analyzed whether our results on diet composition were in line with previous findings on species‐specific primary feeding habitats (as described in Nowald et al., 2018, see Table 4) and found several publications describing cranes using atypical foraging habitats. The large differences in diet composition that we found for black‐necked cranes, common cranes, and sandhill cranes at different study sites reflect the high adaptive ability to land use change of these species. For both species with primary feeding habitat in wetlands and species with primary feeding habitat in non‐wetland areas, we found observations of atypical foraging habitat. While this indicates adaptability of the respective species’ to land use changes, it also reflects a certain dependence on wetland habitat even in cases where the primary feeding habitat is non‐wetland. Sandhill and common cranes mainly forage in non‐wetland areas during staging and wintering. However, our data show high proportions of vertebrate and invertebrate prey in the diet that are derived not only from croplands but also from wetlands during these seasons (Nowald & Fleckstein, 2001, Table 4). Similarly, demoiselle cranes primarily forage in non‐wetland habitats but occasionally also use wetlands, as shown by results on wetland invertebrates in their diet (Sarwar et al., 2013). Our results also demonstrate that species with formerly very distinct wetland foraging habitats have recently also used agricultural areas for foraging. For example, whooping cranes, whose survival in wintering grounds is related to a threshold in the abundance of a single crab species as their main prey item (Pugesek et al., 2013), have been observed feeding on crops in summer (Barzen et al., 2018). The primary foraging source of wintering Siberian cranes is rhizomes of specific water plants, whose occurrence in shallow water is highly dependent on fluctuating water levels (Jiao et al., 2017). An observation of large flocks of Siberian cranes foraging in wet meadows on tubers of a different plant indicates a possible diet shift in response to scarcity of their primary foraging source (Burnham et al., 2017; Jia et al., 2013).

**TABLE 4:** Primary feeding habitat of crane species in the breeding and non‐breeding seasons adapted from (Nowald et al., 2018)

Primary feeding habitat Breeding season	Primary feeding habitat Non‐Breeding Season	Crane species	Verification by Review Results	Number of articles (quantitative/qualitative)	Important references for diet composition
Wetland	Wetland	Siberian crane	t.s.e	0/6	Degtyarev et al. (2013), Degtyarev and Sleptsov (2013), Jia et al. (2013), Chiba (2018)
		Wattled crane	n.a.	0/0	–
Wetland	Wetland & Non‐wetland	Hooded crane	√	0/2	Jing et al. (2002), Jiao et al. (2017)
		Red‐crowned crane	t.s.e.	7/22	Li et al. (2014), Luo et al. (2017), Luo et al. (2015), Jinming et al. (2017)
		Whooping crane	√	2/16	Zimorski et al. (2013), Barzen, Thousand, et al. (2018)
Wetland & Non‐wetland	Wetland & Non‐wetland	Black‐necked crane	√	2/8	Dong et al. (2016), Namgay and Wangchuk (2016), Liu et al. (2014), Li et al. (2009)
		Brolga	n.a.	0/0	–
		Sarus crane	√	0/3	Jha and McKinley (2014)
		White‐naped crane	√	0/3	Lee et al. (2007), Andronova and Bolan (2008)
Wetland & Non‐wetland	Non‐wetland	Black‐crowned crane	n.a.	0/0	–
		Common crane	t.s.e.	5/18	Zhan et al. (2007), Avilés et al. (2002), Abrar et al. (2017)
		Sandhill crane	t.s.e.	11/30	Krapu et al. (2014), Ballard and Thompson (2000)
		Gray‐crowned crane	√	1/1	Gichuki (2000)
Non‐wetland	Non‐wetland	Demoiselle crane	t.s.e.	0/1	Sarwar et al. (2013)
		Blue crane	n.a.	0/0	–

Note

This classification was compared with this review´s information on diet composition: √= the review results report cranes feeding in the species’ primary feeding habitat of the respective season; t.s.e. = the review results report cranes feeding in other habitats than the species’ primary feeding habitat of the respective season; n.a. = no articles were identified in the review.

Similar to whooping and Siberian cranes, natural foraging habitats of red‐crowned cranes are different types of shallow wetlands, where they feed on fish and amphibians. Accordingly, our results show a share of more than 35% animal‐derived food for red‐crowned cranes at all study sites (Table 3). However, as natural wetland habitat is degraded and natural prey is decreasing (Abrar et al., 2017; Jinming et al., 2017; Luo et al., 2017; Yang et al., 2015), the proportion of plants such as maize and rice grains in the diet of red‐crowned cranes has increased. This can partly be explained by supplementary food (i.e., maize and crucian carp) provided to red‐crowned cranes as a compensatory measure at the degraded sites (Ke et al., 2009; Li et al., 2014; Luo et al., 2015).

### 4.3 Types of crops consumed by cranes

**FIGURE 2:**
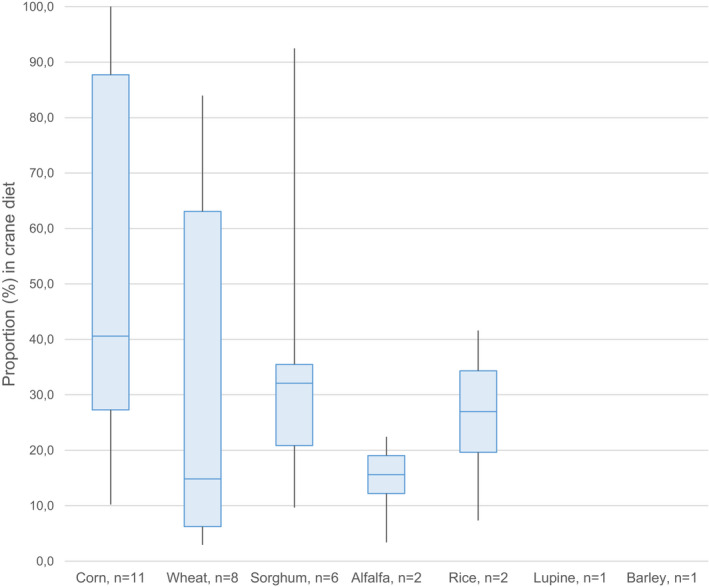
Proportion of crops in the diet of cranes, where *n* = number of results for the specific crop found among 20 publications specifying the type of crop. Lupine and barley were identified as components of the crane diet, but their proportions in the diet were not analyzed

Maize was found most frequently and with the highest share in the diets of all crane species, followed by wheat and sorghum (*Sorghum bicolor* Linnaeus Moench). The crude protein content of all identified crops ranged between 8 and 20% of the dry weight (Figure 2). All 15 crane species are omnivorous, and their digestive system is not adapted to digest high‐fiber plant foods. Thus, they are dependent on invertebrate and vertebrate prey and plants with low fiber, such as berries, tubers, and seeds (Nowald et al., 2018). In accordance with this general pattern, a study by Zou et al. (2012) revealed that red‐crowned cranes selected plants with a high content of crude protein and a low content of crude fiber. However, crude protein may not be the only factor affecting food choice by cranes, for example, biochemicals in unprocessed soybeans prevent the assimilation of nutrients in the digestive system of cranes and soybeans are not used by cranes despite their high level of crude protein (Krapu et al., 2004). In general, our review of diet composition indicates that cranes show high adaptability to forage on different kinds of agricultural crops depending on their availability in the landscape. For example, the largest part of the diet of demoiselle cranes during spring migration consists of chickpea (*Cicer arietinum* L.), wheat, and sorghum, whereas chickpea was replaced by mustard (*Brassica campestris* L.) during the autumn migration. In that case, the use corresponds to the seasonal variation in the availability of the different crops (Sarwar et al., 2013). Wintering common cranes in Spain selected newly sown cereal grain over highly available green plant material but avoided germinated cereal seeds. Consequently, the share of crops in their diet was high upon their arrival at the site in early November (i.e., when the sowing of winter grain had just finished) and decreased throughout the winter (Avilés et al., 2002) (see Table 3).

## 5 CONCEPTUAL FRAMEWORK OF AGRICULTURE‐CRANE INTERACTIONS

**FIGURE 3:**
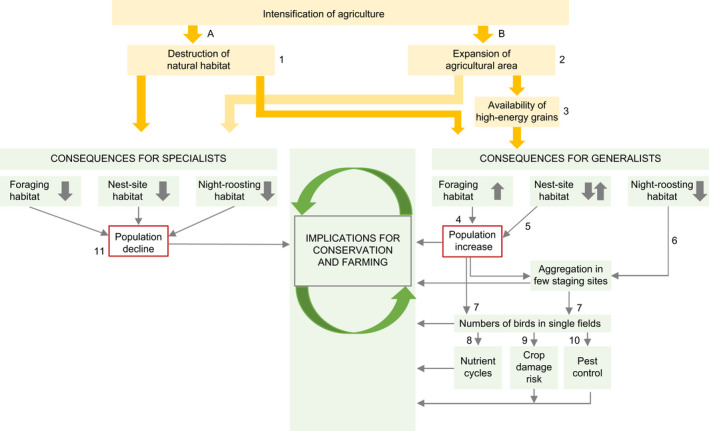
Conceptual framework of two main impact pathways of agricultural intensification on crane species: (1) Expansion of agricultural area destroys natural habitat (see section III.1.a.i); (2) Expansion of agricultural area creates foraging habitat (see section III.1.a.ii); (3) Agricultural practices influence the availability of high‐energy cereal grain in the landscape (see section III.1.a.iii); (4) Improved foraging opportunities in agricultural areas is a factor affecting population growth (section III.1.b.v); (5) Protected nest‐site habitat is a necessary condition for population growth; (6) Along migratory flyways, night‐roosting habitat is limited (Pearse et al., 2010); (7) Increased population sizes and limited availability of staging sites lead to high numbers of cranes in individual fields; (8) High concentrations of birds impact the site nutrient cycle (see section III.1.c.iii); (9) High concentrations of birds raise the risk for substantial crop damage (see section III.1.c.i); (10) When foraging on insects, cranes can have a positive effect on pest control (see section III.1.c.ii); (11) Loss of wetland habitat leads to decreasing populations of crane species dependent on this habitat (see Appendix S1)

Our review reveals that all crane species rely on agricultural crops to varying degrees and are therefore also differently affected by changes in land use, agricultural crops, and technology. The influence of agriculture on crane species can be summarized by two main pathways: destruction of natural habitats and provision of cereal grain (Figure 3): Depending on each species’ ability to adapt to changes in their natural habitat, crane populations develop in opposing directions, and intervention measures should be implemented accordingly. For species that are dependent on wetlands as foraging grounds (see Table 4), the expansion of agriculture decreases key habitats (i.e., foraging, nest‐site, and night‐roosting habitats), and populations decline accordingly as for example Siberian, wattled cranes, and red‐crowned cranes (see Appendix S1). The population of whooping cranes is currently increasing despite its dependence on wetland habitat due to the prohibition of hunting and protection of critical habitat within the migratory corridor (Smith, 2019). Cereal grain in agricultural landscapes affects the availability of high‐energy foraging sources for cranes but the effect on population level has not yet been well studied (see section III.1.b.v). However, apart from whooping cranes and the resident population of red‐crowned cranes (which are fed throughout the year in Hokkaido, Japan (Masatomi et al., 2007), all species that are currently increasing (i.e., sandhill, common, and demoiselle cranes) forage extensively in agricultural areas (Table 3). A precondition for positive population growth of cranes is the protection of nest‐site habitat, an aim of the Natura2000 network in Europe (Nilsson et al., 2019; Rozylowicz et al., 2019) and local and binational schemes for conservation of wetland habitats in North America (Saunders et al., 2019). The lack of protection of nest‐site habitats is a likely factor contributing to the decline of some crane species despite their ability to use agricultural areas for foraging (i.e., brolga, black‐crowned, gray‐crowned, white‐naped, black‐necked, and sarus cranes). Shallow wetlands are critical not only as nest‐site habitat for cranes but also as night‐roosting habitat at staging sites during migration. For sandhill and common cranes, the number of suitable staging sites along flyways is limited (Mirande & Harris, 2019). For example, the Platte River in Nebraska is used for spring staging by 600,000 sandhill cranes, which accounts for more than 70% of their total population (Krapu et al., 2019). As the degradation of river channels has limited the availability of roosting habitats, large numbers of cranes congregate in fields in the area (Krapu et al., 2014; Vogel et al., 2013). The staging sites of common cranes in Europe often show similarly large population of 20,000–200,000 individuals (AG Kranichschutz Deutschland, 2017; Ligue pour la Protection des Oiseaux, 2021). A large congregation of cranes at a specific staging site may have negative effects not only on agricultural production but also on the local nutrient flows and transmission of pathogens (see sections III.1.c.iii and III.1.c.iv). Other ecosystem effects were not identified in the reviewed publications.

## 6 DISCUSSION

### 6.1 Winners and losers of land use change

It is increasingly acknowledged that research needs to go beyond the analysis of mechanisms within a species’ natural habitat and adopt a landscape‐scale approach to better understand population processes (Tscharntke et al., 2012). Responses to land use change vary between and within species functional groups depending on whether the species are able to adapt to the changed conditions in the landscape (Martins et al., 2020). In our review, we find support for previous and general findings that generalist species are less affected and can actually also benefit from intensified (i.e., in our case agricultural) land use while specialist species decline, become displaced or become restricted to remnant natural habitats (Devictor et al., 2008).

### 6.2 Wetland destruction and decreases in specialist crane species

While the protection of wetland habitat is crucial for most crane species, the negative effect of wetland destruction is stronger for species that only marginally use non‐wetland habitats for foraging (i.e., Siberian, red‐crowned, and wattled cranes; see section III.3). The limited available results on diet composition suggest that these species also rely on animal prey to a larger extent than other cranes (see Table 4). Previous research has pointed to the critical dependence of cranes on natural wetland habitat. Three out of the four crane species classified as endangered are highly or very highly dependent on wetlands (i.e., Siberian, red‐crowned, and whooping cranes), whereas those that are less dependent on wetlands (low to moderate) are the only four crane species with a conservation status of least concern (i.e., brolga, demoiselle, sandhill, and common cranes; Harris & Mirande, 2013). Our results also align with evidence for other bird species, showing that generalists dominate bird communities in agricultural landscape (Devictor et al., 2008). In addition to species‐specific physiological abilities to adapt to specific foraging sources, the time species have had to adapt could also be another important factor. Agricultural land use has historically been a main driver of the drainage of natural wetlands in North America, Europe, and Australia (Millennium Ecosystem Assessment (Program) 2005), whereas the same process is a relatively recent phenomenon in many parts of Asia, Africa, and South America (Austin et al., 2019). This may also imply that species in different parts of the world may have had different time frames for adapting to the new conditions. Our results support that generalist crane species in Europe and North America (i.e., demoiselle cranes, common cranes, and sandhill cranes) are well adapted to foraging in agricultural areas. However, some crane species in tropical regions (i.e., gray‐crowned, black‐crowned, and sarus cranes) also show adaptation to conversion of natural habitat by foraging and nesting in agricultural areas (see section III.1.b). This contrasts with the general pattern in tropical regions where many generalist species are not able to persist in areas, where the remaining natural habitat is converted to agricultural areas (Batáry et al., 2020). The decrease in crane species in tropical regions is likely triggered by other factors, such as the destruction of nest sites, the collection of chicks for international trade, illegal hunting, and intentional and unintentional poisoning (Mirande & Harris, 2019; Olupot, 2016).

### 6.3 Generalist crane species have high adaptability to land use change

For sandhill and common cranes, our results show large variation in diet composition in different regions (Table 3), indicating that these species are opportunistic and have a great ability to adapt to changes in foraging habitats. Moreover, the population development of generalist crane species is positively affected by agricultural food sources (common, sarus, sandhill and blue cranes; see section III.1.b.v), and the number of cranes using a certain staging point is influenced by the availability of food in agricultural areas in the surroundings (section 3.1.b.iv). In line with our results, foraging in agricultural areas has been identified as a major factor in the population growth of several other wildlife species, such as the recent population increase in several European goose species (*Anatidae*) due to a shift in habitat utilization from natural grassland and wetlands to pasture and cropland (Fox & Abraham, 2017; Fox & Madsen, 2017) and the recent population growth of wild boar (*Sus scrofa* Linnaeus) due to agricultural intensification (Massei et al., 2015).

### 6.4 Population growth of generalist crane species and possible ecosystem effects

Competition between “winners” and “losers” of land use change may be an important driver of biotic homogenization; that is, the species that adapt well to newly created habitats may suppress specialist species in the remaining natural habitat patches. For example, a study on bird communities in a landscape of vineyards and oak woodland remnants showed that the species that were adapting well to vineyard expansion may have a negative impact on species associated with adjacent natural habitats (Muñoz‐Sáez et al., 2020). Similar patterns may be expected for increasing generalist crane species foraging on vast resources in agricultural areas but sharing breeding and roosting habitats with other, potentially more vulnerable, bird species. Moreover, the effects of nutrient transport to natural habitats caused by wildlife foraging in agricultural areas are generally not well understood (Tscharntke et al., 2012). We have summarized how crane staging sites can affect nutrient flow in surrounding soil and waterways (section III.1.c.iii). These effects can be so severe that it is recommended to limit the number of staging cranes to secure the trophic state of natural water bodies (Litaor et al., 2016b). They are an example of spillover effects of agricultural areas to natural habitat patches, which have been increasingly studied in other species (Bell & Tylianakis, 2016; Blitzer et al., 2012; Boesing et al., 2021; van Schalkwyk et al., 2020).

## 7 MANAGEMENT IMPLICATIONS

### 7.1 Multi‐objective management of agricultural landscapes

It is critical to acknowledge the differential responses of species to future land use changes in order to implement effective conservation measures (Martins et al., 2020; Pardini et al., 2009). We have summarized evidence on species‐specific variation in crane foraging habitat (section III.2.b) as well as on the variation in their response to agricultural land use changes (section III.1.b). This suggests that the conservation of cranes requires a combination of land sparing and land sharing at several spatial scales (Ekroos et al., 2016; Grass et al., 2019), in line with recommendations of the 2019 IUCN Crane Conservation Strategy (Mirande & Harris, 2019). Especially for habitat specialists among crane species, it is highly important to protect existing natural wetland habitat and restore lost wetlands (i.e., land sparing; see section 3.3; Ekroos et al., 2016). However, protected areas are criticized for failing to halt biodiversity loss, as the land available is not sufficient to sustain viable wildlife populations (Crespin & Simonetti, 2019). This shortcoming is especially severe for crane species because they use a diverse set of separated habitat patches within a landscape and along their migratory flyways. Therefore, it is crucial to integrate agricultural areas into crane conservation planning, as agreed upon in the 2010 Convention on Biological Diversity (Aichi Target 7) (i.e., land sharing) and also in international partnerships for the conservation of waterbird habitat (Patterson‐Abrolat et al., 2018). As land sharing and sparing are not mutually exclusive, the growing knowledge on crane habitat use (section III.1.b) could be a valuable resource for identifying how large an area of natural or semi‐natural habitat is needed and how these habitat patches should be distributed across the landscape (Tittonell et al., 2020). Policies in agricultural landscapes need to consider the multiple sometimes diverging objectives of crop production and biodiversity conservation. This has to be done in view of the complex interactions within the agro‐ecological system. For instance, the Common Agricultural Policy (CAP) in the EU and the Farm Bill Conservation Title Programs in the United States have both promoted the use of cover crops, which are sown after harvest in late summer and left until spring to prevent drainage of nutrients and protect against soil erosion (Tittonell et al., 2020). While benefiting soil fertility and the species richness of the soil biota, these policies are an incentive that reduces the area and time of stubble field availability for crane foraging, potentially raising the risk of crop damage to autumn‐sown crops. Our review has summarized several strategies used to prevent crop damage by cranes (see section III.1.d.iii). However, research on bird pests of agricultural crops and the efficacy of control techniques has focused on the management of non‐threatened species (Bomford & Sinclair, 2002; Fox et al., 2017). While there is growing knowledge on management of cranes on cropland available in the grey literature (Austin et al., 2018; Harris, 2010; Mitchusson, 2003), documentation and studies of crop damage by cranes in scientific literature are scarce. Nevertheless, our results show that the strategies currently used for crop protection against cranes have several limitations. Like other large grazing birds, cranes quickly habituate to various scaring techniques, and scaring often just displaces them to neighboring fields (Austin & Sundar, 2018; Nilsson et al., 2018; Vegvari & Tar, 2002; Van Velden et al., 2016). To prevent this, it is recommended to use alternating visual and audible stimuli as experience from goose management has shown (Austin & Sundar, 2018; Fox et al., 2017) and to combine scaring with lure crops or diversionary feeding sites (Nowald, 2018; Végvári & Hansbauer, 2018). In addition to its effectiveness in crop protection, diversionary feeding is rated positively because feeding sites are popular tourist attractions (Nevard et al., 2019; Patterson‐Abrolat et al., 2018). First results from a staging site in Israel show that numbers of common cranes increased faster at the feeding site than general population growth (Shanni et al., 2018) and concerns have been expressed that attraction of cranes to feeding could have negative effects on crop damage risk in the long term (Austin & Sundar, 2018). Our review`s findings on cranes selection of staging sites and altering times and duration of staging due to forage availability in agricultural areas (see section IV.2.e) support these concerns. Taste deterrents are praised for protecting newly sown cereals while allowing cranes to forage on other foods in the field that might be harmful to the crop (e.g., weed seeds or damaging insect larvae) (Barzen et al., 2020; Lacy, 2018). However, anthraquinone is currently the only substance available for use in South Africa and the United States, and it is criticized for potential carcinogenic effects and lacking data on risks to birds and other non‐target species (Review Report for the Active Substance Anthraquinone of, 2008). Given the efficiency of anthraquinone in preventing crop damage by cranes, further research on its environmental effects or other substances applicable as taste deterrents could be beneficial.

Our conceptual framework analysis (section III.3) has illustrated how aggregations of large numbers of cranes at few available wetland staging sites increase the risk of crop damage in the surrounding agricultural landscape. Based on this problem and the above‐mentioned limitations of current crop protection strategies, we propose that crop protection measures be coordinated not only at the scale of affected fields or staging sites but at the scale of migratory flyways. Cross‐boundary cooperation in the management of wetlands needs to go beyond borders of protected sites and actively involve stakeholders in adaptive management of crane population numbers and available foraging habitats in agricultural areas (Nilsson et al., 2016, 2019). For instance, close cooperation with farmers can be used to discuss potential changes in crop rotation or time of harvest time to increase the availability of cereal stubbles during times of crane staging as already implemented by several local initiatives (Elphick et al., 2018; Smirenski et al., 2018). The flyway management plan for pink‐footed goose (Anser brachyrhynchus Baillon) is an example of how scientific evidence can be used to inform collaboration across national borders (Madsen & Williams, 2012). In the long term, it needs to be analyzed whether establishing additional wetland staging sites would dilute damage risk along flyways or rather intensify crop damage problems through a positive effect on crane populations.

### 7.2 Involvement of stakeholders

Our review results suggest that the number of cranes on their fields is a major factor contributing to farmers perceiving cranes as a problem (see section III.1.d.i). This is in contrast to the findings of previous studies on wildlife damage, which have stressed the importance of social factors in triggering conflict and suggested that technical solutions to crop damage are insufficient to resolve conservation conflicts (Dickman, 2010; Peterson et al., 2010). In contrast, cooperation between stakeholders and managing authorities is viewed as critical to reach satisfying trade‐offs between conservation and agricultural objectives, and the role of participatory approaches as well as social learning for inclusion of stakeholders in decision making is highlighted (Crespin & Simonetti, 2019; König et al., 2020; Redpath et al., 2017). The importance of such cooperations is explicitly expressed also in the context of crane conservation (Barzen, 2018; Nilsson et al., 2021). We have summarized accounts of community‐based initiatives that used either the positive cultural image of cranes or a system of price premiums for crops grown in crane habitat to gain farmers´ acceptance of crane conservation (see section III.1.d.iv & vi). A multitude of examples of crane conservation initiatives actively involving stakeholders are described in the grey literature (Austin et al., 2018). Yet in our review, we found research on stakeholder perspectives and policy options to be underrepresented. Less than 10% of articles on agriculture‐crane interactions focused on societal responses, only 9% used the term “conflict,” and only 7% mentioned crop damage as a potential driver of conflict. As grey literature reports on crop damage by cranes prove the rising problems of crop damage by cranes (see Introduction), we conclude that the peer‐reviewed literature lacks a focus on understanding stakeholders’ perspectives in comparison with describing crane ecology, which was the focus of 60% of publications. In wildlife research, "coexistence" describes the goal of humans and wildlife living in shared landscapes, in which both persistence of wildlife populations and social legitimacy and tolerable levels of risk are ensured (Carter & Linnell, 2016). While working toward this goal, it is important to consider that various stakeholder groups in a landscape have different beliefs about ideal population sizes of wildlife as well as about appropriate management. These beliefs are based on internal, psychological variables, behavioral variables (e.g., farmer vs. wildlife manager and past experiences with wildlife), and specific factors of a certain case (e.g., wildlife species and management actions) (Zinn et al., 2000). Consequently, any solution to problems of crop damage created by cranes will be a trade‐off involving increased agricultural productivity, species conservation, and other stakeholder interests (Tittonell et al., 2020).

## 8 STATE OF KNOWLEDGE AND RESEARCH GAPS

For this manuscript, we have analyzed information on cranes and their interactions with the agricultural sector as available in peer‐reviewed scientific publications in English language. We explicitly chose to include only journals listed in Scopus or Web of Science to get an overview of the current discourse within the system of peer‐reviewed scientific literature. In the analyzed publications on both agriculture‐crane interactions and on diet composition, most articles focused on common cranes (23%), red‐crowned cranes (21%), and sandhill cranes (20%), possibly indicating the importance of agricultural areas for these species. The focus on these species may also reflect different available resources for research between regions and a possible bias because of language restrictions (Amano et al., 2021). Nevertheless, the research gap on particularly vulnerable crane species is concerning because our review shows that interactions with agriculture are important for all crane species. Regarding studies on diet composition, the differences in methods and sample size make comparisons of the results less straightforward, as the methods have different advantages and disadvantages (Nielsen et al., 2018). For example, aquatic prey items such as fish are more easily assimilated than plant material, which needs to be taken into account when interpreting the results (Luo et al., 2015). Despite the lack of comparable high‐quality data, our results are still useful for identifying general trends. Yet another problem is that diet composition within single species varies largely across locations as well as across the different stages of the annual cycle (Table 3), but only the diet of sandhill and common cranes has been analyzed at a wide variety of sites. This highlights that crane diet composition constitutes a key research gap especially because recent changes in the foraging habitat of several crane species have been reported (see section III.2.b). Especially for endangered crane species, analyses of foraging habitat and diet composition are essential for identifying and protecting key habitats. Moreover, only 20 articles specified the type of crop consumed by cranes. More detailed information on crop type could be useful not only to inform the discussion of potential crop damage risk but also for the targeted design of crop protection strategies. We have pointed out the lack of research on actual crop damage and the effectiveness of crop damage prevention methods (section IV.2.a) as well as lacking knowledge on stakeholders’ perspectives (see section IV.2 b). As our analysis has shown how crane movements, habitat use, and diet composition are influenced by agricultural practices, the current rates of global agricultural intensification and expansion will necessitate new research on these aspects of crane ecology.

## 9 CONCLUSIONS

1. Most research on agriculture‐crane interactions focuses on crane habitat selection rather than the impact of crane foraging on agricultural crops and management options. We identified two main pathways of agriculture‐crane interactions: (i) The destruction of natural habitat by agricultural expansion and (ii) the provision of cereal grain in agricultural landscapes.
2. The degree to which crane species can adapt to land use changes and use cereal grain as food source may be important factors explaining their population response.
3. Ecosystem effects associated with the second pathway, such as nutrient transfer to natural habitat or competition with other species need to be analyzed in future research.
4. Conservation needs to combine land sparing and land sharing, by preserving and restoring natural wetland habitat for specialist species and by cooperating with farmers to secure forage availability on agricultural areas for generalist species.
5. While research on farmer attitudes toward cranes is scarce, our results suggest that acceptance for cranes and conservation initiatives, in general, is dependent on effective crop protection as much as on stakeholder participation.
6. Reconciliation of crane conservation and agricultural production needs to be implemented from the local to the flyway scale: By using existing crop protection methods, crop damage can be effectively prevented at the local scale. However, to assure long‐term sustainability, effects of agricultural land use changes and the establishment of protected areas on crane migration pattern and population development need to be addressed and managed at flyway scale. International cooperation in the management of major staging sites needs to address the problem of large congregations of cranes at single sites.
7. Acknowledging the dual nature of agriculture‐crane interactions allows us to merge the perspectives of sustainable agricultural production and species conservation. As current agricultural policies provide incentives that contradict the aim of providing foraging area in harvested fields (e.g., the promotion of cover crops), conservation initiatives and agricultural policy should be streamlined to achieve multi‐objective management of crane population sustainability, on the one hand, and the habitat capacity and economic productivity of agricultural landscapes on the other hand.

## CONFLICT OF INTEREST

The authors declare no conflict of interest.

## AUTHOR CONTRIBUTIONS

**Karoline Hemminger:** Conceptualization (lead); Data curation (lead); Formal analysis (lead); Visualization (lead); Writing – original draft (lead); Writing – review & editing (lead). **Hannes König:** Conceptualization (equal); Funding acquisition (lead); Project administration (lead); Supervision (equal). **Johan Månsson:** Conceptualization (equal); Methodology (equal); Visualization (equal); Writing – review & editing (equal). **Sonoko‐Dorothea Bellingrath‐Kimura:** Supervision (equal); Writing – review & editing (equal). **Lovisa Nilsson:** Conceptualization (equal); Methodology (equal); Supervision (equal); Writing – review & editing (equal).

## Supporting information

Appendix S1

Appendix S2

## Data Availability

No original data were generated. The datasets generated as bibliography for the review are available from the corresponding author upon reasonable request.
