# Supplementary material for: Winners and losers of land use change: A systematic review of interactions between the world’s crane species (Gruidae) and the agricultural sector
Source: Ecol Evol. 2022 Mar 24;12(3):e8719. doi: 10.1002/ece3.8719 (PMC8948072; doi:10.1002/ece3.8719)
Supplement: Supplementary file 1 — Appendix S1 [file ECE3-12-e8719-s001.docx]

**Appendix S1:** Summary of status, numbers and key threats for the 15 crane species, adapted from Harris & Mirande, 2013, population numbers taken from Mirande & Harris, 2019

| **Red List Status*** | **Species Name** | **Distribution** | **Estimated**  **Population Size** | **Current Trend** | **Dependence on**  **Aquatic Habitat** | **Main Threats** |
| --- | --- | --- | --- | --- | --- | --- |
| CR | Siberian Crane  *Leucogeranus leucogeranus (Pallas, 1773)* | Asia | **3600-4000** | Decreasing, western subpopulation reduced to a single individual | Very high | Habitat loss, especially due to building of hydro-electric dams |
| EN | Grey Crowned Crane  *Balearica regulorum (Bennet, 1834)* | East and Southern Africa | **26,500-33,500** | Decreasing | Moderate | Habitat loss and fragmentation,  capture for domestication and illegal trade, powerline collisions |
|  | Red-crowned Crane  *Grus japonensis (Müller, 1776)* | Northeast Asia | **2800-3430** | Increasing in Japan, continental population stable, but decreasing in China | High | Degradation of breeding and wintering sites |
|  | Whooping Crane  *Grus americana*  *(Linnaeus, 1758)* | North America | **689** | Increasing | High | Habitat loss, hydrological changes to wintering habitat, power line collisions, small population size |
| *VU* | Blue Crane  *Anthropoides*  *paradiseus*  *(Lichtenstein, 1793)* | South Africa,  Namibia | **25,000-30,000** | Increasing | Low | Habitat loss due to mining and agriculture, grassland conversions  power line collisions, windfarms, capture for trade |
|  | Black-necked Crane  *Grus nigricollis*  *(Przevalski, 1876)* | Central Asia | **10,000-10,200** | Increasing | Moderate | Grassland degradation, drying up of marshes, partly due to climate change and intensification of agricultural practices |
|  | Black Crowned Crane  *Balearica pavonina*  *(Linnaeus, 1758)* | West Africa, Sudan, Ethiopia | **43,000-70,000** | Decreasing | Moderate | Habitat loss and degradation due to drought, wetland drainage, industrial and dam construction, hunting and capture for illegal trade |
|  | Hooded Crane  *Grus monacha*  (Temminck, 1835) | Northeast Asia | **14,500-16,000** | Increasing in Japan and South Korea; decreasing in China | Moderate | Wetland loss and degradation due to building of hydro-electric dams; human disturbance; over-fishing |
|  | Sarus Crane  *Antigone antigone*  *(*Linnaeus, 1758) | South Asia, Australia | **15,000-20,000** | Stable or decreasing | Moderate | Drainage of wetlands for conversion to agriculture, hunting, collection of eggs for trade and to limit damage to crops |
|  | Wattled Crane  *Bugeranus carunculatus*  *(Gmelin, 1789)* | East and Southern Africa | **9000-10,000** | Probably decreasing | High | Loss and degradation of wetlands due to river regulation, intensified agriculture and mining; nest disturbance, grass-burning regimes, capture for illegal trade |
|  | White-naped Crane  *Antigone vipio (Pallas, 1811)* | Northeast Asia | **7000-7800** | Decreasing | Moderate | Loss of wetlands due to agricultural expansion, growing demand for water and building of hydroelectric dams; hunting; human disturbance |
| LC | Brolga  *Antigone rubicunda (Perry,1810)* | Australia, New Guinea | **50,000-100,000** | Unknown, decreasing in parts of its range | Low to moderate | Habitat loss due to agricultural expansion |
|  | Demoiselle Crane  *Anthropoides virgo (Linnaeus, 1758)* | Eurasia, some winter in Africa | **170,000-220,000** | Probably decreasing | Low | Habitat loss and degradation from agriculture, hunting for sport and intentional poisoning to prevent crop damage |
|  | Common Crane  *Grus grus*  *(Linnaeus, 1758)* | Eurasia, some winter in Africa | **>700,000** | Increasing | Low to moderate | Habitat loss and degradation through dam construction, urbanisation and agricultural expansion; nest disturbance, hunting and illegal shooting |
|  | Sandhill Crane  *Antigone canadensis*  *(Linnaeus, 1758)* | North America, Northeast Siberia | **827,000** | Increasing | Low to moderate | Habitat loss due to changing agriculture, water management, collisions with powerlines, crop damage prevention |
