# Supplementary material for: Winners and losers of land use change: A systematic review of interactions between the world’s crane species (Gruidae) and the agricultural sector
Source: Ecol Evol. 2022 Mar 24;12(3):e8719. doi: 10.1002/ece3.8719 (PMC8948072; doi:10.1002/ece3.8719)
Supplement: Supplementary file 2 — Appendix S2 [file ECE3-12-e8719-s002.pdf]

Interaction	Explanation (corresponding section)	Crane species															Key references
		Black crowned crane	Black-necked crane	Blue crane	Brolga	Common crane	Demoiselle crane	Grey crowned crane	Hooded crane	Red-crowned crane	Sarus Crane	Sandhill crane	Siberian crane	Wattled crane	White-naped crane	Whooping crane	
Destruction of natural habitat	Expansion of agriculturally used areas leads to draining of wetlands and a decrease of other natural habitat (III.1.a.i)		x	x		x		x	x	x	x	x	x	x	x	x	Pearse et al. 2018; Peng et al. 2018; Fakarayi et al. 2016; Zhou et al. 2016; Kuhn et al. 2011; Liu et al. 2008; Avilés 2004
Habitat improvement	Expansion of agricultural areas also creates foraging habitat for those species that are adapted to feed on agricultural crops or grassland (III.1.a.ii)			x							x						Nevard 2019; Van Velden et al. 2017; Mc Cann et al. 2007
Influence on food availability	How much agricultural forage is available to cranes is dependent on the choice of crops in an area, as well as harvesting and soil cultivation techniques (III.1.a.iii)					x			x	x		x			x		Sherfy et al. 2011; Anteau et al. 2011; Pearse et al. 2010; Krapu et al. 2004; Guzman et al. 1999; Zhan et al. 2007
Pesticide effects	Inadequate use of pesticides can reduce natural forage available to cranes, deliberate poisoning of cranes with pesticides causes death of individuals (III.1.a.iv).			x						x	x		x				Lu et al. 2007; Krapu et al. 2004; Mukherjee et al. 2002

Interaction	Explanation (corresponing section)	Crane species															Key references
		Black crowned crane	Black-necked crane	Blue crane	Brolga	Common crane	Demoiselle crane	Grey crowned crane	Hooded crane	Red-crowned crane	Sarus Crane	Sandhill crane	Siberian crane	Wattled crane	White-naped crane	Whooping crane	
Foraging on permanent pastures	Cranes use agricultural grasslands as foraging grounds (III.1.b.i)				x	x			x	x		x	x				Franco et al. 2000; Fakarayi et al. 2016
Foraging on arable land	Cranes use arable land as foraging grounds (III.1.b.i)		x	x	x	x	x	x	x	x	x	x	x	x	x	x	Liu et al. 2010; Wu et al. Liu et al 2010; McCann et al. 2009; McCann et al. 2000; Zou et al. 2006; Minton et al. 2003; Nilsson et al. 2019; Vegvari et al. 2002; Wu et al. 2017
Breeding in agricultural areas	Crane species have adapted to breeding sites in paddy fields or agricultural grasslands (III.1.b.ii)						x	x			x						Korovin et al. 2011; Gopi Sundar 2011; Gopi Sundar 2011; Borad et al. 2002; Borad et al. 2001
Roosting and foraging in aquaculture/ paddy fields	Crane species use artificial wetlands such as aquaculture and paddy fields for roosting and foraging (III.1.b.iii)					x			x	x			x		x	x	Lu et al. 2006; Lee et al. 2007; Fujioka et al. 2010; Wood et al. 2010; Zhou et al. 2010
Habitat selection in response to changing agriculture	Availability of agricultural forage in a landscape influences selection of staging and wintering sites as well as the time of migration (III.1.b.iv)					x					x					x	Aviles 2004; Belair et al. 2014; Leito et al. 2008; Kraou et al. 2004; Aviles et al. 2002; Guzman et al. 1999; López Saut et al. 2011, Mireles et al. 2017
Population development influenced by availability of agricultural forage	High amounts of agricultural forage available decreases foraging efforts by cranes and thus increases their population viability (III.1.b.v)			x			x				x	x					Van Velden et al. 2017; Nevard et al. 2019; Korovin 2011; Lacy et al. 2015

Interaction	Explanation (corresponding section)	Crane Species															Main References
		Black crowned crane	Black-necked crane	Blue crane	Brolga	Common crane	Demoiselle crane	Grey crowned crane	Hooded crane	Red-crowned crane	Sarus Crane	Sandhill crane	Siberian crane	Wattled crane	White-naped crane	Whooping crane	
Crop damage	When cranes feed on newly sawn seeds or trample part of a field for building a nest, this creates crop damage (III.1.c.i).			x	x	x			x	x	x	x	x				Van Velden et al. 2016; Nevard et al. 2018; Montràs-Janer et al. 2019; Borad et al. 2001
Foraging on pest insects	Reports on incidences of cranes foraging on pest insects, which is benefitial to agricultural production (III.1.c.ii)					x					x						Singh 2010; Nowald 2001
Effects on nutrient cycles	Especially at larger staging sites, defacation by birds influences nutrient cycles in the agro-ecosytem (III.1.c.ii)					x						x					Navedo, 2015; Litaor, 2014; Litaor, 2015
Transmission of pathogens	Cranes have been reported as carriers of pathogens, threatening the safety of agricultural products and creating risks for poultry farms	x				x						x					Gardner, 2011; Bello, 2008
Crop damage as a driver of conflict	Crop damage is referred to as a driver of conflict (III.1.d.i)			x	x					x		x					Van Velden et al. 2016; Laubhan and Gammonley 2001; Nevard et al. 2018; Cullen 2010; John et al. 2003
Famers positive attitude	In some cultures, farmers explicitly have a positive attitude towards cranes (III.1.d.ii)			x	x				x		x					x	Kim et al. 2011; Gopi Sundar et al 2011; Muheebwa-Muhoozi 2001
Crop protection	Methods used to prevent crop damage by cranes, such as scaring, diversionary feeding or taste repellents (III.1.d.iii)				x	x				x	x	x			x		Barzen et al. 2020; Van Velden et al. 2016; Montras-Janer 2019
Compensation payments/financial incentives	Direct payments to farmers to compensate economic losses or to support beneficial agricultural practices; income from ecotourism; marketing of products grown in crane habitat (III.1.d.iv)					x	x	x	x	x	x		x		x		Montras-Janer 2019; Khai and Yabe 2015; Kim et al. 2011
Adapted agricultural management	Changes in crop rotation or timing of harvest that are favorable to cranes and/or reduce the risk of crop damage (III.1.d.v)		x		x	x				x	x	x					Sherfy et al 2011; Krapu et al. 2004; Cullen 2011
Generating Awareness and Stakeholder Participation	Recommendations to use educational programs and/ or to seek active participations of farmers in crane conservation (III.1.d.vi)										x						Kaur et al. 2008; John et al. 2003
